# Behavioural and Socioeconomic Drivers of Household Food Waste in Ireland: Evidence for Targeted Food Policy

**DOI:** 10.3390/foods15162920

**Published:** 2026-08-20

**Authors:** Courage Yaw Krah, Paul Hynds, Anushree Priyadarshini

**Affiliations:** 1 Sustainability and Health Research Hub, Technological University Dublin, D07 EWV4 Dublin, Ireland; couragekrah@gmail.com (C.Y.K.); paul.hynds@tudublin.ie (P.H.); 2 School of Business, Maynooth University, W23 WK26 Maynooth, Co. Kildare, Ireland

**Keywords:** household food waste, behavioural interventions, date labelling, quantile regression, Ireland, consumer behaviour

## Abstract

Households have been reported to account for more than a third of food waste (FW) generated in the most developed and high-income countries. Despite this, there remains a limited understanding of the behavioural and sociodemographic factors driving household food waste (HFW) generation in most countries, including the Republic of Ireland (ROI). This study therefore examined how behavioural practices and sociodemographic characteristics independently and interactively influence HFW generation in the ROI. Data from a national online survey (*n* = 966 households; 3163 individuals) were analysed using hierarchical quantile regression. Households with children (Mixed households) reported the highest HFW (1177 g/household/week), and single-adult households recorded the highest per capita median waste (females = 596.0 g/cap/week and males = 496.5 g/cap/week). Behavioural factors were stronger predictors than sociodemographic characteristics. Reliance on date labels was the strongest predictor of HFW, associated with an additional 239 g/week. Each additional weekly shopping trip was associated with 140 g/week more waste, whereas using a shopping list was associated with 42 g/week less waste. Some effects were moderated by sociodemographic factors: reliance on date labels was more strongly associated with waste among adults aged 18–24, and shopping frequency more strongly associated with waste in middle-income households. The findings point to the value of reforming date labelling, supporting more planned shopping, and encouraging the active reuse of leftovers, with attention to differences across household groups. The findings also have direct implications for Ireland’s obligations under SDG 12.3 and the EU Waste Framework Directive.

## 1. Introduction

Food waste (FW) is a pressing global issue with far-reaching implications for food security, environmental sustainability, and economic efficiency. The United Nations Environment Programme’s (UNEP) Food Waste Index Report 2024 estimates that approximately 1.05 billion tonnes of food were wasted globally at the retail and consumer stages in 2022, amounting to an economic loss of approximately $1 trillion [1]. These losses intensify food insecurity in a world where nearly 783 million people continue to experience chronic hunger [2], while simultaneously accounting for 8% to 10% of annual global greenhouse gas emissions [3].

The associated resource costs are equally substantial. Approximately 250 km^3^ of freshwater and 1.4 billion hectares of agricultural land are used each year to produce food that is discarded, highlighting the scale of avoidable inefficiency in contemporary food systems [1].

Across the food supply chain, the magnitude of waste generated at the household level is particularly concerning. Globally, private households account for approximately 60% of all food discarded at the retail and consumer stages, amounting to some 631 million tonnes annually [1]. A similar pattern is evident within the European Union, where households generate more than half of total food waste (53%), estimated at approximately 30 million tonnes per year, equivalent to 69 kg per capita [4,5]. In the Republic of Ireland (ROI), the Environmental Protection Agency (EPA) reported that households discarded approximately 221,000 tonnes of food in 2023, representing 26% of national food waste generation (835,000 tonnes), imposing an estimated economic burden exceeding €1.3 billion [6]. The environmental implications are also significant: Flanagan and Priyadarshini [7] estimated that household food waste in Ireland generates approximately 600,000 tonnes of CO_2_-equivalent emissions each year.

Given the scale and consequences of household food waste (HFW), regulatory frameworks targeting food waste reduction have gained prominence. In September 2025, the European Parliament adopted a revised Waste Framework Directive (Directive (EU) 2025/1892), which later came into force in October 2025. The Directive legally obliges Member States (MS) to reduce per capita food waste at retail and consumption stages, including households and food service operations, by 30% by December 2030, relative to average levels recorded between 2021 and 2023 [8]. This binding commitment is consistent with the United Nations Sustainable Development Goal (SDG) Target 12.3, which calls for a halving of per capita food waste at the retail and consumer levels by 2030 [9]. As a Member State, the ROI has further committed to a 50% reduction in food waste by 2030 through its National Food Waste Prevention Roadmap [10]. Within this regulatory landscape, robust national evidence on the factors that drive HFW is essential for designing policies that are both effective and efficient.

As evidenced by the systematic review by Krah et al. [11], a substantial body of research has shown that HFW does not arise from a single, isolated cause. Rather, it emerges from the complex interplay of behavioural, psychological, socio-cultural, and material factors [12,13,14]. Behaviours such as inadequate meal planning, impulse purchasing, overbuying, and poor food storage have been repeatedly associated with higher waste volumes [15,16]. Beyond behavioural drivers, sociodemographic characteristics including age, income, household composition, and dietary patterns have also been identified as contributing factors, though their effects vary considerably across settings and contexts [17,18].

In seeking to explain both the mechanisms and the relative contributions of these drivers, scholars have drawn upon a range of theoretical frameworks. One of the most widely applied is the Theory of Planned Behaviour (TPB) [19], which emphasises the roles of attitudes, subjective norms, and perceived behavioural control in shaping consumer behaviour, including food waste generation. Another frequently employed model is the Motivation–Opportunity–Ability (MOA) framework [20], which adopts a broader perspective by recognising that behaviour is shaped not only by motivation, but also by situational opportunities and individual capabilities. These frameworks, however, were not originally developed with food waste in mind and therefore carry certain inherent limitations. The TPB, for instance, is primarily concerned with psychological intentions and may inadequately account for the routine, habitual, and practice-based dimensions that characterise much of household food waste behaviour [14,21]. Despite these constraints, both frameworks laid important conceptual groundwork for more recent models specifically adapted to the study of consumer food waste.

**Figure 1:**
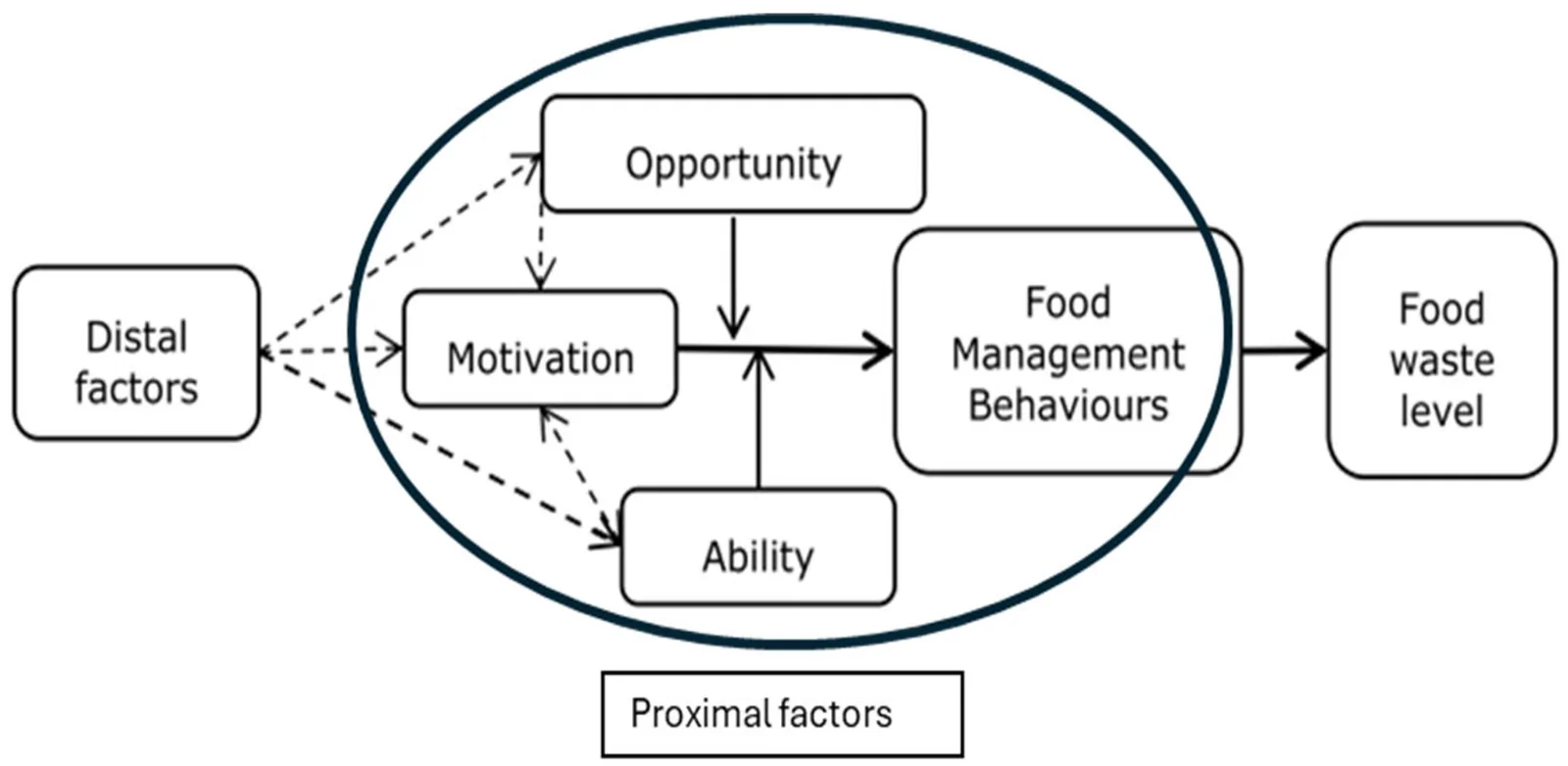
Consumer Food Waste Framework (adapted from van Geffen et al. [22]).

Building on this theoretical lineage, the Consumer Food Waste Framework (CFWF), developed by van Geffen et al. [22], has emerged as one of the most contextually attuned models in the field and serves as the analytical foundation for the present study. Adapted from the MOA framework, the CFWF (Figure 1) conceptualises the drivers of consumer food waste across two levels: distal and proximal factors. Sociodemographic characteristics such as household size, age, and income constitute the distal factors, shaping the structural and contextual conditions within which consumers manage food on a daily basis. The proximal factors, in turn, encompass motivation, opportunity, and ability, as well as the household food management behaviours that mediate the relationship between these individual capacities and actual waste generation.

Within this proximal sphere, the framework proposes that motivation, ability, and opportunities are mediated through daily food management routines, such as meal planning, cooking, and leftover management, which represent the immediate behaviours most directly associated with food waste generation. This distinction carries important policy implications. While behavioural pathways are generally more amenable to intervention, their effectiveness depends on how well policies are tailored to the demographic and socioeconomic contexts in which they are implemented [23].

Where HFW behaviour has been examined, behavioural and sociodemographic predictors are often analysed either in isolation or as equivalent variables within single-stage models, rather than through systematic hierarchical approaches capable of capturing their additive and interactive effects [15,18]. Consequently, understanding of how food waste behaviours vary across sociodemographic groups remains limited. Without such analytical rigour, policy interventions risk misallocating resources towards populations for whom relevant behavioural effects are weak, absent, or substantially different. Addressing this requires household-level data in which behavioural practices, household structure, and sociodemographic characteristics are measured together. The ROI provides such a setting. Existing Irish studies have focused primarily on estimating food waste quantities, with comparatively limited attention to the mechanisms underlying generation and how these vary between households [6,7]. National estimates also provide a benchmark against which this behavioural evidence can be positioned.

Against this background, the present study aimed to achieve three objectives. First, it quantified HFW volumes and the composition of food categories across household types. Second, identified the relative and additive contributions of behavioural practices to weekly household food waste generation. Third, it examined how sociodemographic characteristics moderate behavioural drivers in order to identify priority groups for targeted intervention.

Although the study is situated in the Irish context, the findings are expected to hold wider relevance for comparable high-income countries, where household structures, food systems, and consumption patterns are broadly similar. The evidence generated directly supports Ireland’s obligations under SDG 12.3 and the revised EU Waste Framework Directive [8].

## 2. Materials and Methods

### 2.1. Survey Design, Data Collection and Harmonisation

A cross-sectional questionnaire was developed based on a review of existing literature and relevant previous national surveys [24,25]. The questionnaire comprised 40 questions across four main sections, namely: (1) demographic and contextual factors, including age, gender, income, employment status, household composition, and residential location; (2) dietary habits and food management practices, such as diet type, shopping frequency, food expenditure, use of shopping lists, expiry date monitoring, cooking frequency, and leftover management; (3) self-reported food waste, covering weekly quantities and types of discarded food (e.g., vegetables, meat, dairy); and (4) attitudes and motivations towards FW, including reasons for discarding food, motivations to reduce waste, and disposal methods (e.g., general bin, composting, feeding to pets). In line with van Herpen et al. [25], participants reported their HFW volumes on a weekly basis, using familiar household measures such as bowls, glasses, handfuls, and ladles. To facilitate accurate estimation, the questionnaire included visual aids such as photographs of food items and portion sizes.

A pilot version of the questionnaire was developed and hosted online for two weeks, during which 20 participants took part and provided feedback regarding the clarity of wording, question relevance, response options, and survey length. The insights from this phase were used to refine and improve the final version of the questionnaire. The finalised questionnaire was hosted online on the SurveyMonkey platform from April 2023 to August 2024. Eligibility criteria for participation required respondents to be at least 18 years old, reside in the ROI, and possess sufficient awareness of the volume of FW generated in their household. Respondents who indicated a lack of knowledge about their HFW were excluded from further participation.

A multimodal approach was used to reach a broad, diverse audience. The survey was shared through email invitations, institutional websites, and social media platforms such as X (formerly Twitter), Facebook, Instagram, and LinkedIn. Additionally, QR codes linking to the survey were printed on posters and distributed at public events and community spaces. This comprehensive outreach strategy ensured that the study captured perspectives from individuals across different socioeconomic backgrounds throughout Ireland. Approximately 1250 responses were received. Incomplete responses were deleted, yielding a final analytic sample of 966 households with complete data on all relevant variables.

Given that FW volumes were recorded using familiar household units, it was necessary to standardise these measurements by converting them into gram equivalents, as shown in Table 1 [25]. To enhance the precision of these conversions and ensure their applicability within the context of the ROI, the research team conducted an additional validation process by weighing representative sample portions of various food items corresponding to the measurement units used in the questionnaire (e.g., ladles, pints, and packs). The final study questionnaire is presented as a Supplementary File.

### 2.2. Estimating per Capita Waste Generation from Total Household Food Waste

To facilitate more accurate comparisons of FW volumes across households of different sizes and compositions, HFW was standardised to a per capita basis. Respondents were first categorised into three household structures: single-adult households, multi-adult households (adults without children), and mixed households (households including children) [26]. Each household structure was further grouped according to the number of occupants (e.g., one, two, three, and four or more persons).

For single-adult households, the reported household FW equalled the individual’s weekly per capita FW. As the household and individual FW volumes were equal in single-adult households, per capita FW was also reported separately for males and females. For multi-adult and mixed households, HFW was standardised by converting total weekly household FW to per capita values by dividing it by the number of occupants in the household [1]. These estimates were subsequently summarised by household structure and household size.

To account for the influence of household size in subsequent analyses, household FW was residualised following the approach described by García et al. [27]. Expected household FW was estimated by regression (median regression) of total household FW (g/household/week) on household size. Median regression was used because the distribution of household FW is right-skewed and to ensure consistency with the second-stage model. The model fit results and diagnostics are included in the Supplementary File (Table S1). Predicted household FW attributable to household size was estimated, and residual FW values were calculated as the difference between the observed household FW and the predicted value. These residuals represent the variation in household FW that cannot be explained by household size. Consequently, residual FW values were used as the dependent variable in the subsequent quantile regression analyses, enabling the effects of behavioural and sociodemographic predictors to be examined independently of household size.

### 2.3. Statistical Analysis and Modelling

All statistical analyses were conducted using SPSS (Statistical Package for the Social Sciences) software, version 29.0.2. Before analysis, preprocessing steps were applied to ensure data integrity. Categorical variables were converted to their binary form using one-hot encoding. Exploratory data analyses were conducted to characterise the outcome variable (residual HFW) and its association with the predictor variables. Associations with continuous independent variables were assessed using Spearman’s rank correlation, while Kruskal–Wallis tests and the Mann–Whitney U test were used for categorical independent variables (Tables S2 and S3 included in the Supplementary File). For modelling, the residuals (the difference between the observed and expected FW volumes) were used as the primary outcome variable. As the residuals indicated positive skewness, quantile regression (median regression) was selected. Beyond the observed non-normality, this model was chosen because it imposes no distributional assumption on the outcome, is robust to the outliers and heteroscedasticity characteristic of self-reported FW data, and models the conditional median, which represents the centre of a strongly right-skewed distribution more meaningfully than the conditional mean.

Model development followed a hierarchical block-entry framework similar to that reported by Hynds et al. [28]. The independent variables (Table 2) were delineated into six conceptual hierarchies based on their likely roles in HFW generation [29]. The full wording of each questionnaire item, its response format, coding, units, and reference category, where applicable, is provided in the Supplementary file (Table S6). Continuous predictors are expressed in their original units, and all categorical predictors were dummy-coded against the reference categories indicated. Variables were also entered based on their distal–proximal blocks as derived from the Consumer Food Waste Framework (CFWF). Accordingly, model specification was guided by a priori conceptual reasoning, ensuring that behavioural, motivational, and sociodemographic predictors were assessed within their respective theoretical domains.

**Table 2:** Classification of factors influencing household food waste generation.

Psychological & Behavioural Determinants	Sociodemographic Determinants
Best before date waste	Sex
Spoiled food waste	Age
Leftover-induced waste	Income
Excess cooking waste	Education level
Storage space-induced waste	Occupation
Economic motivation reduction	Ethnicity
Time-saving motivation reduction	Dietary pattern
Environmental motivation reduction	Season
Cultural motivation reduction	Urban/rural setting
Ethical motivation reduction	Province
Mixed waste disposal	
Composted food waste	
Shopping frequency	
Grocery spending	
Shopping list usage frequency	
Lunch preparation frequency	
Dinner preparation frequency	
Non-home meal consumption frequency	
Leftover saving frequency	
Leftover reuse frequency	

The regression model began with two base specifications: a primary-effects model and an interaction-only model (Tables S3 and S4 included in the Supplementary File). This layered approach enabled the identification of both additive (main) and multiplicative (interaction) influences on weekly HFW generation. Variable entry into the models followed a forward stepwise conditional approach, with variables considered for inclusion if their significance level was *p* < 0.05, flagged for review if *p* was between 0.05 and 0.10, and automatically removed if *p* > 0.10 [28]. Stepwise selection operated only within these pre-specified blocks. This approach recognises that the conceptual framework identifies the domains relevant to HFW generation but not which indicator within each domain is most strongly associated with waste, while restricting the search space relative to unconstrained stepwise selection. Candidate main effects are listed in Table 2. Interactions were not searched exhaustively. Candidates were restricted to behavioural predictors retained as main effects, crossed with age, household income, or settlement type on theoretical grounds. The full set of interaction terms tested is reported in Table S5.

To quantify each hierarchy’s contribution to model performance, the incremental change in pseudo-R^2^ relative to the preceding hierarchy was calculated. Prior to model estimation, multicollinearity among predictors was assessed using variance inflation factors (VIF), and all included predictors had VIF below 5. Model fit was reported as Koenker and Machado’s pseudo-R^2^, calculated as the proportional reduction in the sum of weighted absolute deviations relative to the null model, and predictive accuracy was measured as Mean Absolute Error (MEA). As a robustness check, the final model was re-estimated at the 25th and 75th percentiles to establish whether the associations observed at the median hold across other quantiles.

Forward stepwise conditional selection retained only independently contributing predictors, limiting multicollinearity; four of the ten ranked reason and motivation items were retained. Estimates were stable across the main-effects (Table S4), interactions-only (Table S5), and full models with similar magnitude and direction.

## 3. Results

### 3.1. Sociodemographic Composition of the Study Sample

The survey sample (966 households, ≈3163 individuals) was predominantly middle-aged, with individuals aged 35–54 accounting for about 53% of participants. As shown in Table 3, households with children were the most surveyed household type (56%), and a total of 80% of respondents together resided in multiperson households (multi-adult-only households and households with children and adults). Socioeconomic indicators exhibited relatively affluent and highly educated participants. Nearly one-third of respondents (29%) reported annual household incomes exceeding €100,000, while 77% had attained at least an undergraduate education level (42% undergraduate; 34% postgraduate). Geographically, 61% of the sample lived in urban areas, with 42% residing in Dublin County. The ethnic composition was predominantly Irish (61%). Employment was primarily full-time (70.7%), and dietary profiles were largely omnivorous (70.0%), although approximately 30% reported adherence to alternative diets (flexitarian, vegetarian, or vegan).

### 3.2. Food Waste Volumes and Composition Across Household Types

**Figure 2:**
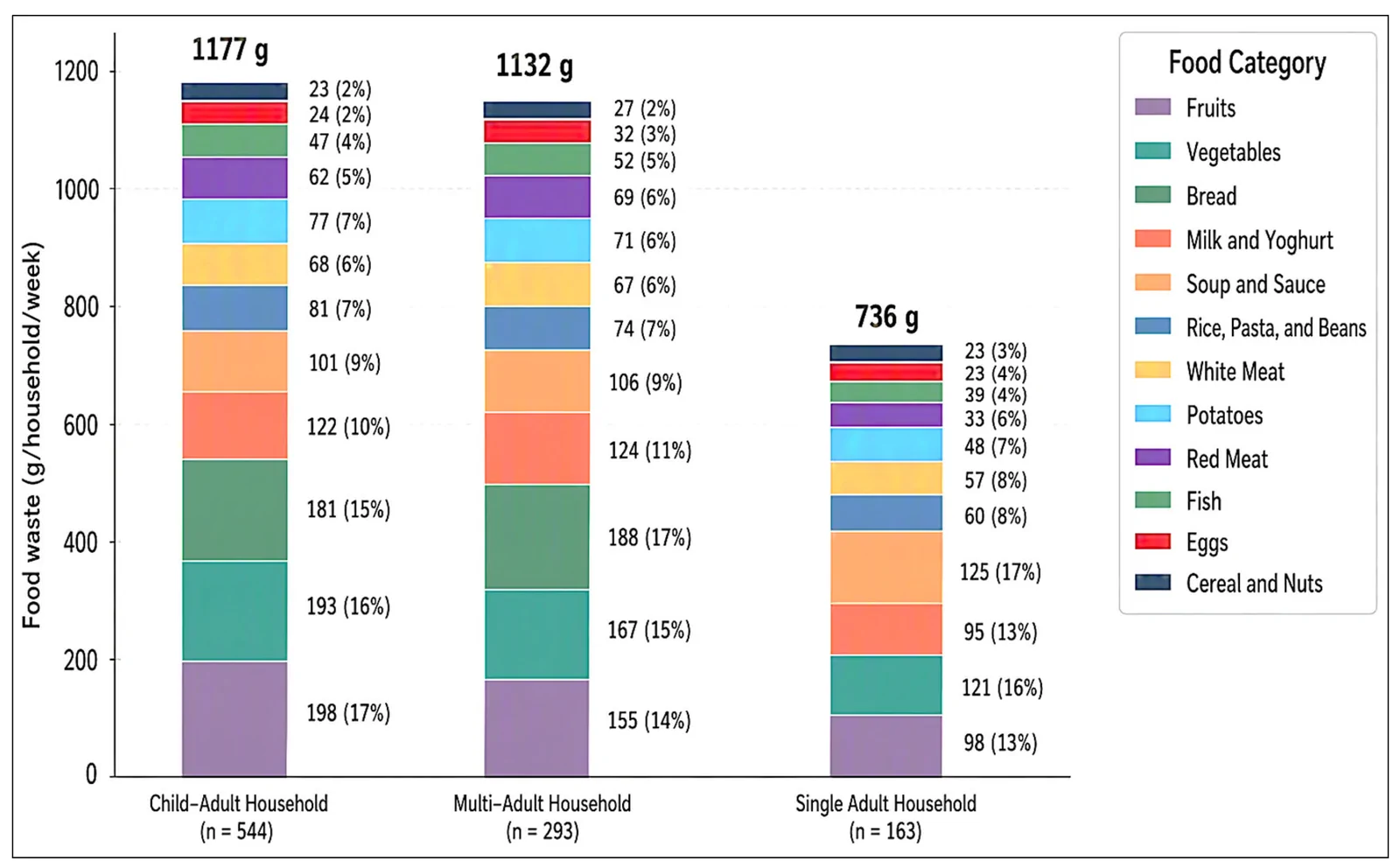
Volumes of various food items wasted across different household types.

Mean weekly FW across all households was 1080 g/week, with vegetables (16%), fruits (16%), and bread (15%) accounting for the largest proportions of total FW by mass (Figure 2). Mixed households recorded the most waste (1177.3 g/week), followed by multi-adult households (1132.5 g/week) and single-adult households (736.2 g/week). In mixed households, fruits (16.9%), vegetables (16%), and bread (15%) were the main items discarded by mass. In multi-adult households, bread accounted for the largest share of discarded food items (17%), followed by vegetables (15%) and fruits (14%). In single-adult households, milk and yoghurt (17%) and vegetable waste (17%) accounted for the largest share of FW, followed by bread (13%).

**Figure 3:**
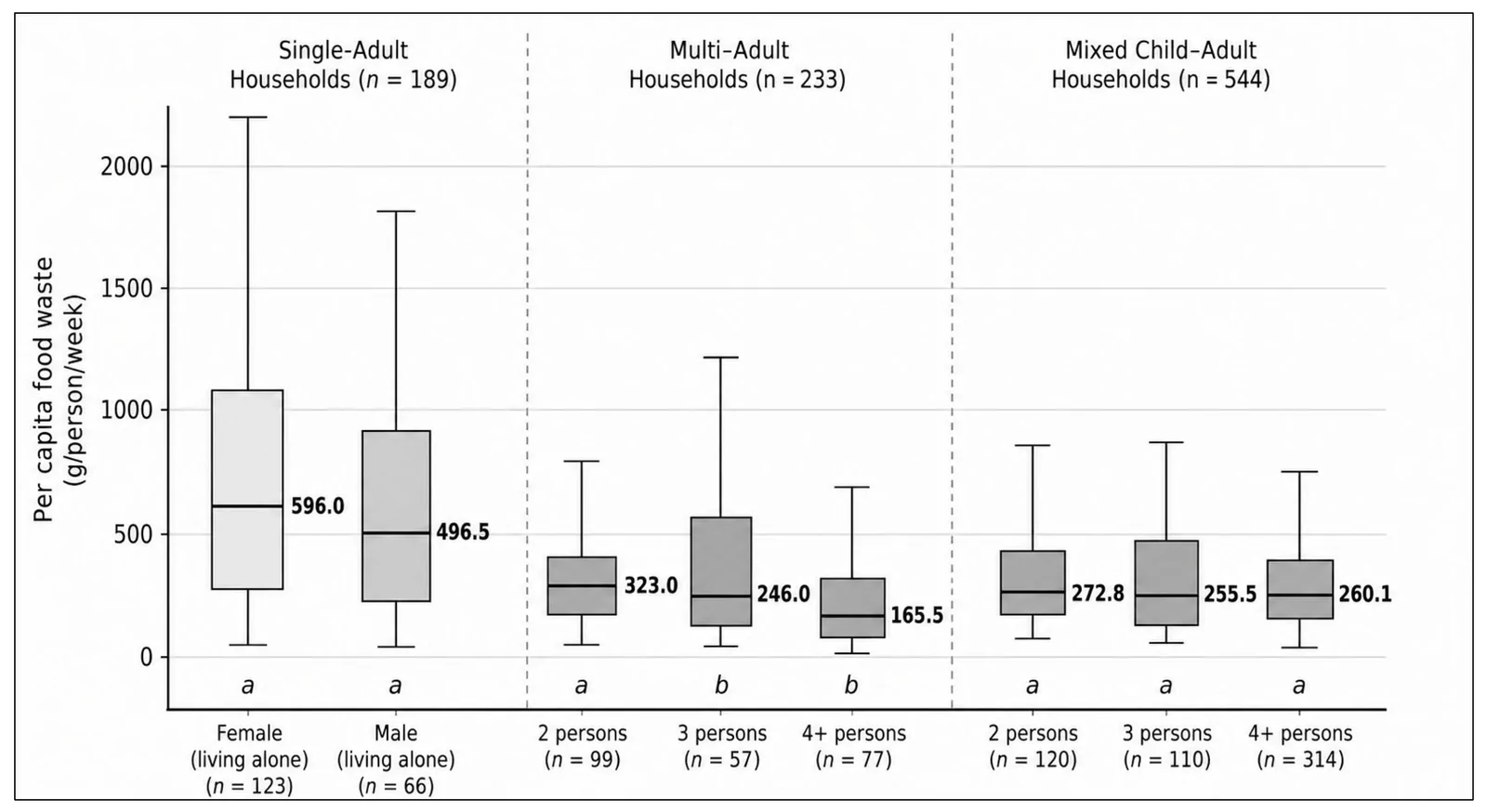
Per capita estimates of household food waste across household structures.

On a per capita basis (Figure 3), females living alone (in single-adult households) recorded higher median FW volume (596.0 g/person/week) than males living alone (496.5 g/person/week), although the difference was not statistically significant (Mann–Whitney U = 4523.5, *p* = 0.196). In multi-adult households, median per capita FW declined with household size, from 323.0 g/person/week in two-person households to 165.5 g in households of four or more (Kruskal–Wallis H(2) = 18.34, *p* < 0.001). Dunn–Bonferroni comparisons indicated that two-person households generated significantly more per capita FW than both three-person (*p* = 0.047) and four-or-more-person households (*p* < 0.001), while the latter two did not differ (*p* = 0.525). Among mixed child–adult households, median per capita FW was comparatively stable across household sizes (272.8, 255.5 and 260.1 g/person/week respectively), with no significant differences detected (H(2) = 2.26, *p* = 0.323).

### 3.3. Hierarchical Quantile Regression Modelling of Residual Household Food Waste

The residuals showed positive skewness (1.72) and kurtosis (3.69), and the Shapiro–Wilk test indicated significant deviation from normality (W = 0.79, *p* < 0.001), consistent with the use of median regression. The final hierarchical model (Table 4) achieved a pseudo-R^2^ of 0.478, a modest improvement in fit over the main-effects model (0.450), with a mean absolute error of 299 g/week.

**Table 4:** Hierarchical quantile regression results predicting weekly household food waste generation.

Variables	B	SE	Sig.	95% CI
***Conditions Prompting Discard***	***MAE = 435.52; R^2^ = 0.239***			
Best before date waste	238.80	8.45	<0.001	[222.21, 255.39]
Food waste due to spoilage	93.08	8.69	<0.001	[76.02, 110.14]
***Motivational Factors***	***MAE = 400.03; R^2^ = 0.301***			
Economic motivations	−94.28	9.25	<0.001	[−112.43, −76.13]
Time-saving motivations	−124.25	9.17	<0.001	[−142.24, −106.26]
***Shopping Behaviours***	***MAE = 352.31; R^2^ = 0.384***			
Shopping frequency	140.06	13.23	<0.001	[114.09, 166.03]
Individual grocery spending	6.37	0.72	<0.001	[4.96, 7.78]
Shopping list use frequency	−41.87	9.97	<0.001	[−61.44, −22.31]
***Meal Management***	***MAE = 346.96; R^2^ = 0.394***			
Non-home meal frequency	60.72	16.71	<0.001	[27.93, 93.51]
Leftover reuse frequency	−42.15	12.09	<0.001	[−65.87, −18.43]
Leftover saving frequency	42.62	12.43	0.001	[18.23, 67.02]
***Disposal Methods***	***MAE = 319.97; R^2^ = 0.441***			
General mixed waste	6.09	0.52	<0.001	[5.07, 7.10]
Organic waste bin usage	1.83	0.50	<0.001	[0.85, 2.80]
***Sociodemographics***	***MAE = 314.33; R^2^ = 0.451***			
*Dietary patterns (Ref: Omnivorous)*				
Vegan diet	−116.32	55.21	0.035	[−224.66, −7.98]
***Interaction Terms***				
***Best before date waste × Age (Ref: 45–54 years)***	***MAE = 311.49; R^2^ = 0.456***			
18–24 yrs × Best before date waste	32.90	12.74	0.010	[7.90, 57.89]
***Food waste due to spoilage × Household income (Ref: > €100,000)***	***MAE = 310.39; R^2^ = 0.458***			
€20,000–€40,000 × Food waste due to spoilage	20.77	9.83	0.035	[1.48, 40.06]
€80,000–€100,000 × Food waste due to spoilage	21.86	10.73	0.042	[0.81, 42.91]
***Shopping frequency × Household income (Ref: > €100,000)***	***MAE = 300.79; R^2^ = 0.474***			
€20,000–€40,000 × Shopping frequency	121.03	37.46	0.002	[47.5, 194.6]
€80,000–€100,000 × Shopping frequency	89.06	36.13	<0.001	[18.1, 160.0]
***Leftover reuse frequency × Age*** (Ref: 45–54 years)	***MAE = 298.92; R^2^ = 0.478***			
18–24 yrs × Leftover reuse frequency	−54.48	27.59	0.049	[−108.6, −0.3]
25–34 yrs × Leftover reuse frequency	−77.59	22.04	<0.001	[−120.8, −34.3]
55–64 yrs × Leftover reuse frequency	−96.80	27.99	<0.001	[−151.7, −41.9]

*B = coefficient; SE = standard error; MAE = mean absolute error; R^2^ = pseudo-R^2^(Koenker and Machado); CI = confidence interval*.

Reasons for discarding food accounted for approximately 50% of the fit achieved by the full model(pseudo-R^2^ = 0.239), with discarding food due to best-before dates associated with a 239 g/week (*p* < 0.001) increase in median residual FW. Spoilage-related discards also emerged as a significant (*p* < 0.001) predictor, contributing 93 g/week of FW per event in a given week. This effect was further moderated by income: households earning €20,000–€40,000 produced an additional 21 g/week (*p* = 0.035) of FW due to food spoilage, and those in the €80,000–€100,000 bracket produced an additional 22 g/week (*p* = 0.042), compared to those earning over €100,000. The association between date labelling and food waste was moderated by age, though only among the youngest respondents: households aged 18–24 discarded an additional 33 g/week (*p* = 0.010) relative to the reference group, while the corresponding terms for the 25–34 and 35–44 groups were not significant.

Factors that motivate people to reduce HFW generation contributed an additional 12.8% ((0.301 − 0.239)/0.478) to improving model fit, with economic motivation associated with a 94 g/week (*p* < 0.001) reduction and time-saving motivation with a 124 g/week (*p* < 0.001) reduction in median residual FW. The addition of shopping behaviours further improved the model fit by 16.7% (0.384). Each additional shopping trip within a given week was associated with a 140 g increase in FW (*p* < 0.001), with this effect particularly pronounced in the €20,000–€40,000 income group (+121 g/week, *p* = 0.002).

Meal management strategies produced a smaller improvement in fit, raising pseudo-R^2^ from 0.380 to 0.394 (2.9%). Saving leftovers more frequently was paradoxically associated with a 43 g/week increase in food waste (*p* = 0.001), whereas reusing leftovers was associated with a 42 g/week reduction (*p* < 0.001). Age moderated the benefits of leftover reuse, with reductions most pronounced among households aged 56–65 (−97 g/week, *p* < 0.001), followed by those aged 26–35 (−78 g/week, *p* < 0.001) and 18–25 (−54 g/week, *p* = 0.049).

Disposal methods raised pseudo-R^2^ from 0.394 to 0.441.(10.5%). Mixed waste bin usage was associated with 6 g/week more FW (*p* < 0.001), and organic bin use with 2 g/week more (*p* < 0.001). Finally, the inclusion of sociodemographic characteristics and their interaction terms increased the pseudo-R^2^ by 7.1% (from 0.441 to 0.478). In the base model (Tables S4 and S5), sociodemographic factors such as dietary pattern, household income, education, and ethnicity were statistically significant predictors (*p* < 0.05). However, in the final model, only age and income retained significance through interaction effects, indicating that their influence was primarily moderating rather than direct. Several of the behavioural variables were also significant in the main effects model but not in the interaction models.

## 4. Discussion

This study provided novel insights into HFW generation in the ROI by examining total and per capita FW across different household types. The study also highlighted how household demographics and consumption behaviours shaped waste outcomes. The sample reflected the national population in some key demographic characteristics. In terms of household composition, 80% of respondents lived in multiperson or family households compared with 80% nationally [30], and urban–rural residence (61% urban, 39% rural) closely matched the 2022 Census distribution (63% and 37%, respectively). However, the sample was more affluent and more highly educated than the general population, with 29% reporting annual household incomes above €100,000 and 77% holding at least an undergraduate qualification. Dublin residents were also over-represented, comprising 41.7% of the sample compared with 28% nationally [30].

### 4.1. Food Waste Volumes

This study found that participating households discarded an average of 1080 g of food waste per week, equivalent to approximately 56 kg per household annually. However, it is important to note that self-reported estimates tend to underestimate actual food waste. Therefore, the volumes reported in this study are more likely to represent a conservative estimate of actual household food waste rather than an overestimate.

The findings revealed that the most discarded food items are perishable goods, particularly fruits, vegetables, and bread. This pattern was consistent across all household types and aligned with observations made in earlier national surveys and other high-income countries [10,11,31]. The prevalence of these items in the waste stream has been reported to be driven mainly by both physiological mechanisms and consumer perceptions. Physiologically, the high moisture content in fruits and vegetables accelerates microbial activity, ultimately leading to spoilage [32]. In addition, a previous report by Kubíčková et al. [33] identified the relatively lower cost of these fresh produce and bread as factors that encourage stockpiling and, subsequently, spoilage. This is particularly relevant in the Irish context, where fruits, vegetables, and bread are among the most frequently purchased items, often promoted in bulk offers. Nováková et al. [34] also noted that economic accessibility, particularly in higher-income households, increases the risk of underutilisation and eventual disposal.

As might be expected, calculated per capita FW tended to decrease with increasing household size, suggesting that larger households benefit from economies of scale in food consumption and subsequent FW generation, with shared meals and coordinated shopping reducing surplus and spoilage. This also aligned with previous findings by Oláh et al. [35] and Elimelech et al. [36], who found that food is consumed more efficiently in multiperson households, with less waste, than in single-person households. Conversely, smaller households, particularly those comprising one or two persons, often struggle with portion control and managing perishable goods, as standard food packaging sizes are rarely suited to their needs [37]. These inefficiencies contributed to higher waste levels among smaller households, a trend clearly observed in the present study. Given that nearly one in five Irish households consists of a single adult [30], this demographic represents a critical target for food waste reduction strategies.

### 4.2. Sociodemographic and Behavioural Predictors of Household Food Waste

The developed quantile regression model revealed that the largest share of explained variance was derived from underlying consumer decisions regarding food discard at home, food shopping, and meal management, with sociodemographic variables and their interactions providing additional explanatory power.

Households that ranked best-before dates as a more important reason for discarding food reported 239 g more waste per week. This reliance on best-before dates to discard food suggests misunderstanding of date labelling, a problem extensively documented in previous studies [38,39]. The findings of both Lu et al. [40] and Kavanaugh and Quinlan [38] showed that consumers in the United States of America (USA) misinterpreted best-before dates on packaged food items as warnings about food safety rather than its quality. Lu et al. [40] concluded that effective reform requires both standardised labels and targeted consumer education. The finding further supports the EU proposal for ‘Look–Smell–Taste’ (LST) labelling, which encourages consumers to assess food quality using sensory cues rather than relying solely on date labels [41].

Shopping frequency was another significant predictor, with each additional trip associated with an excess of 140.1 g/week in FW. This aligned with both Cicatiello et al. [42] and Principato et al. [43], who documented that frequent, unplanned shopping leads to impulse purchases and subsequent overstocking. In contrast, planned purchasing, as reflected in the regular use of a shopping list, was associated with reduced waste (−41.9 g/week), consistent with evidence from Jörissen et al. [37] and Stancu et al. [44], both of whom reported that pre-structured shopping facilitates better inventory control. The contrast between saving and reusing leftovers is one of the study’s most practically important findings. While actively reusing leftovers was associated with 42.2 g/week less waste, saving them for later was associated with 42.6 g/week more (*p* = 0.001). This suggests that storing leftovers without a clear plan for consumption may delay rather than prevent disposal. Panda et al. [45], in their study on leftover repurposing, similarly emphasised that the waste-reduction benefits of leftovers depend not on storage alone, but on their active reintegration into later meals. Without planned reuse, saved leftovers remain vulnerable to being forgotten or spoiled.

Motivational factors were also associated with lower waste. Households reporting economic motivations recorded 94.3 g/week less, and those reporting time-saving motivations 124.3 g/week less. These results affirm the findings of Principato et al. [43] that behavioural intentions are often shaped by convenience and financial considerations. The finding also supports an established principle in behavioural economics that practical, self-motivated incentives are often more effective at driving behaviour change than abstract environmental or ethical appeals [46]. The greater effect of time-saving motivation relative to economic motivation suggests that food management practices perceived as convenient or effort-reducing may be found more attractive and readily adopted. This also aligns with findings by Paroissien et al. [47] in France, where the opportunity cost of time was identified as a key predictor of efficient household food use.

Income also moderated the relationship between spoilage-related waste and shopping frequency, with middle-income (€20,000–€40,000) and upper-middle-income (€80,000–€100,000) households showing significantly higher levels of spoilage waste than other groups. This trend supports the dual argument presented by Secondi et al. [17]: lower-income groups may be constrained by storage or budgetary limitations, while higher-income households are more prone to over-purchasing perishables. These findings reinforced the importance of tailoring interventions to reflect both scarcity and surplus. Interestingly, dietary patterns also revealed meaningful differences in waste levels. Vegan households, for instance, generated 116.3 g/week less waste than omnivorous ones, corroborating findings by Schanes et al. [13] and Visschers et al. [48] that plant-based diets tend to reflect more mindful consumption patterns. Promoting sustainable dietary habits may therefore yield additional benefits in waste prevention. Encouraging the adoption of sustainable dietary habits may therefore not only support efforts to prevent FW but also help reduce the environmental impacts associated with unsustainable diet patterns. This dual benefit underscored the importance of promoting sustainable diets as a strategy for both reducing waste and protecting the environment [49].

### 4.3. Limitations and Future Research

While this study provides valuable insights into the drivers of HFW in the ROI, it is not without limitations. First, reliance on self-reported FW volumes may introduce biases, as participants may underreport or inaccurately estimate their waste due to social desirability bias or recall errors [25,50]. Although the use of visual aids and standardised measurement units aimed to improve accuracy, these methods may not entirely eliminate reporting discrepancies, and the estimates presented here are more likely to understate than to overstate actual waste. Second, because FW was recorded at household level in multiperson households, per capita values were standardised volumes for comparison across household types. Third, the study’s cross-sectional design limits the ability to track changes in waste patterns over time. Fourth, the sample was recruited through a self-selected online panel and was more affluent, more highly educated, and more concentrated in Dublin than the national population; surveys of this kind also tend to attract more environmentally conscious individuals [51]. Also, while variable selection was constrained to theoretically pre-specified blocks, the number of interaction terms evaluated increases the risk of Type I error. The reported interaction effects should therefore be considered exploratory and require confirmation in independent samples. Finally, the study focused primarily on behavioural and demographic factors, with limited exploration of external influences such as retail practices, food packaging, or policy frameworks, which could also significantly impact HFW.

Future research should address these limitations through longitudinal designs, objective waste measurement methods such as diaries or waste audits, nationally representative sampling, and broader examination of the structural and commercial factors shaping household food provisioning. Comparative studies could also be undertaken to assess the generalisability of these findings to other high-income countries with similar household structures and food systems.

## 5. Conclusions

This study shows that household food waste is shaped by the interaction between behavioural practices, household structure, and sociodemographic factors, rather than by isolated predictors. Another novel contribution of this study is the demonstration that behavioural effects on food waste are not evenly distributed across the population. The findings highlight how individual motivations and everyday habits operate within broader demographic and socioeconomic contexts to influence food waste generation. Reliance on date labels was more strongly associated with waste among younger respondents, while lower-middle- and upper-middle-income households showed a stronger effect on spoilage and shopping frequency. The waste-reducing effect of leftover reuse was weakest among younger age groups. By incorporating interaction effects, the study provides a more nuanced level of analysis than much of the existing literature.

Taken together, these results point to the need for a shift away from broad, awareness-based campaigns towards targeted, demographically informed strategies that reflect real-world behavioural dynamics. Policy responses should prioritise regulatory reform to clarify and standardise date labelling, behavioural interventions that embed sustainable practices into everyday routines, and targeted education initiatives for high-waste groups, particularly young adults and middle-income households. Aligning interventions with both behavioural drivers and household context is essential for achieving effective and scalable reductions in HFW.

## Figures and Tables

**Table 1:** Reference table for converting food waste units into grams (adapted from van Herpen et al. [25]).

Category	Unit	Estimated Grams
Fresh vegetables	Serving spoon	50
Non-fresh vegetables	Serving spoon	50
Fresh fruit	Piece	100
Non-fresh fruit	Piece	80
Potatoes	Serving spoon	60
Pasta	Serving spoon	50
Rice	Serving spoon	60
Meat	Portion	150
Fish	Portion	150
Sandwich filling	Portion	20
Bread	Slice	35
Bread	Whole bread	800
Cereals	Portion	40
Cereals	Pack	500
Yoghurt et cetera	Portion	150
Yoghurt et cetera	Pack	1000
Cheese	Cube	10
Eggs	Egg	60
Soups	Ladle	150
Soups	Litre	1000
Sauces	Spoon	20
Sauces	Bottle	450

**Table 3:** Sociodemographic and household characteristics of the study sample (N = 966).

Variable	Categories	n	(%)
Age	18–24 years	95	9.83
	25–34 years	197	20.39
	35–44 years	258	26.71
	45–54 years	254	26.29
	55–64 years	116	12.01
	65 years or older	46	4.76
Household income	Less than €20,000	45	4.66
	€20,000–€40,000	157	16.25
	€40,000–€60,000	173	17.91
	€60,000–€80,000	152	15.73
	€80,000–€100,000	163	16.87
	More than €100,000	276	28.57
Household type	Single-adult household	189	19.57
	Multi-adult household	233	24.12
	Household with children and adults	544	56.31
Household composition	Male members	994	33.87
	Female members	1092	37.21
	Children	849	28.93
Household size	1 person	189	19.57
	2 persons	219	22.67
	3 persons	167	17.29
	4 persons	205	21.22
	5 persons	142	14.70
	6 persons or more	44	4.56
Ethnicity	Irish	587	60.77
	Non-Irish White	148	15.32
	African or other Black background	98	10.14
	Asian background	69	7.14
	Mixed ethnicity	64	6.63
Settlement	Predominantly rural areas	377	39.03
	Predominantly urban areas	589	60.97
Education level	Up to vocational level	226	23.40
	Undergraduate degree	408	42.24
	Master’s degree or PhD	332	34.37
Employment	Retired	53	5.49
	Unemployed	61	6.31
	Part-time employment	169	17.49
	Full-time employment	683	70.70
Dietary patterns	Vegan	79	8.18
	Vegetarian	80	8.28
	Flexitarian	131	13.56
	Omnivorous	676	69.98
Province	Ulster	37	3.83
	Connacht	67	6.94
	Munster	228	23.60
	Leinster (excluding Dublin)	231	23.91
	Dublin	403	41.72

## Data Availability

The data presented in this study are available on reasonable request from the corresponding author. The data are not publicly available due to ethical and privacy considerations, as they contain information obtained from research participants under informed consent agreements. In addition, portions of the dataset are currently being used in ongoing research projects by members of the research team.

## Supplementary Materials

The following supporting information can be downloaded at https://www.mdpi.com/article/10.3390/foods15162920/s1, File S1: Study questionnaire. Table S1. First-stage residualisation model and residual diagnostics (N = 966). Table S2. Bivariate correlations between household food waste drivers and weekly waste volumes. Table S3. Weekly household food waste volumes by sociodemographic, geographic, and dietary factors in the Republic of Ireland. Table S4. Hierarchical quantile regression results predicting weekly household food waste generation (Base Model 1; main effects). Table S5. Hierarchical quantile regression results predicting weekly household food waste generation (Base Model 2; interactions only). Table S6. Behavioural and psychological predictors: item wording, response options, and coding. Figure S1. Interaction Coefficients with 95% Confidence Intervals Plot.

## Abbreviations

Abbreviation	Definition
CFWF	Consumer Food Waste Framework
CSO	Central Statistics Office
EC	European Commission
EPA	Environmental Protection Agency
EU	European Union
FAO	Food and Agriculture Organisation
FW	Food waste
GHG	Greenhouse gas
HFW	Household food waste
IPCC	Intergovernmental Panel on Climate Change
MAE	Mean absolute error
MOA	Motivation–Opportunity–Ability (framework)
MS	Member State(s)
REFRESH	Resource Efficient Food and dRink for the Entire Supply cHain (EU project)
ROI	Republic of Ireland
SDG	Sustainable Development Goal
SPSS	Statistical Package for the Social Sciences
TPB	Theory of Planned Behaviour
UNEP	United Nations Environment Programme
USA	United States of America
VIF	Variance Inflation Factor
