# Supplementary material for: Behavioural and Socioeconomic Drivers of Household Food Waste in Ireland: Evidence for Targeted Food Policy
Source: Foods. 2026 Aug 20;15(16):2920. doi: 10.3390/foods15162920 (PMC13512863; doi:10.3390/foods15162920)
Supplement: Supplementary file 1 [file foods-15-02920-s001.zip › Study questionnaire.pdf]

Survey instrument

Questionnaire

We would like to invite adults who are 18 years or older and currently living in the Republic of Ireland to participate in our study. Conducted by Courage Krah, Dr., Dr. Paul Hynds, and Dr. Anushree Priyadarshini from TU Dublin. The study aims to explore volumes, habits, and the environmental and economic impacts of household food waste. It will require approximately 10 minutes to complete this survey, and your participation is completely voluntary; you can withdraw at any point. All information provided will be kept strictly confidential, and no *personally identifiable data will be disclosed in any published materials.*

For any inquiries, please contact Courage at d21128848@mytudublin.ie

A household is defined as a single person or group of people who regularly reside together in the same accommodation and who share the same catering arrangements.

Which one best describes you?

☐ *I live alone and am willing to report on how I handle and dispose of food at home*

☐ *I live in a household with other people and can report on how we collectively handle and dispose of food at home*

☐ *I live in a household with other people, but I can only report on my personal food handling and disposal*

Thanks again for participating! Let's learn a little more about you.

Household demographic information (multi household) (Filter)

When it comes to food-related responsibilities in your household, which role(s) do you fulfill?
Please select all that apply:

☐ *Grocery shopping*
☐ *Meal preparation*
☐ *Food storage and organisation*
☐ *Meal planning*
☐ *Food waste management*
☐ *Other (please specify):* _______________
☐ *None of the above*

Which one of the following best describes the composition of your household?
(Adult = 18+, Children = <18 years)

☐ *1 adult aged 65+*
☐ *1 adult aged <65*
☐ *2 adults, at least 1 aged 65+*
☐ *2 adults, both aged <65*
☐ *3 or more adults*
☐ *1 adult with children under 18*
☐ *2 adults with 1-3 children under 18*
☐ *Other (please specify):* _______________

Please enter the total number of people in your household that fall within the following groups (including you):

Adult Male(s): _____
Adult Female(s): _____
Children: _____
Others: _____

How old are you (years)?

☐ *17 or younger*

☐ 18-24
☐ 25-34
☐ 35-44
☐ 45-54
☐ 55-64
☐ 65-74
☐ 75 or older

Which of the following do you identify with?

☐ Male
☐ Female
☐ Other
☐ Prefer not to answer

Please choose which one best represents your total annual income (before tax):

☐ No income
☐ Less than €10,000
☐ €10,000 - €20,000
☐ €20,000 - €30,000
☐ €30,000 - €40,000
☐ €40,000 - €60,000
☐ €60,000 - €80,000
☐ €80,000 - €100,000
☐ More than €100,000
☐ Prefer not to answer

Kindly select the race/ethnicity that best describes you:

☐ Irish
☐ Any other White background (Non-Irish White)
☐ Asian Irish
☐ Asian background
☐ African/Other Black background
☐ Other (please specify): _______________
☐ Prefer not to answer

Which of the following best describes your current occupat

Which county in Ireland do you currently live in?

Do you consider where you live to be more rural or urban in nature?

☐ More urban
☐ More rural
☐ I'm not sure

Do you live within walking distance to the nearest pub, bar, or café?

☐ Yes
☐ No
☐ I don't know

What is the estimated walking time to the nearest pub, bar, or café from your home?

☐ 1 to 5 minutes (0 to 0.5 km)

☐ *6 to 15 minutes (0.6 to 1.5 km)*
☐ *16 to 30 minutes (1.6 to 2.5 km)*
☐ *31 to 45 minutes (2.6 to 4 km)*
☐ *46 minutes to an hour (4.1 to 5 km)*
☐ *More than an hour*
☐ *I don't know*

Approximately how long will it take to get to the closest pub, bar, or café from your home by driving?

☐ *Less than 15 minutes (0 to 12 km)*
☐ *16 to 30 minutes (13 to 25 km)*
☐ *31 to 45 minutes (26 to 38 km)*
☐ *46 minutes to an hour (39 to 50 km)*
☐ *More than an hour*

Food disposal

Reflecting on the past week, please estimate the amounts of food discarded in your home. This includes food items composted/ recycled, poured down the sink, or fed to pets

Vegetables and Fruits

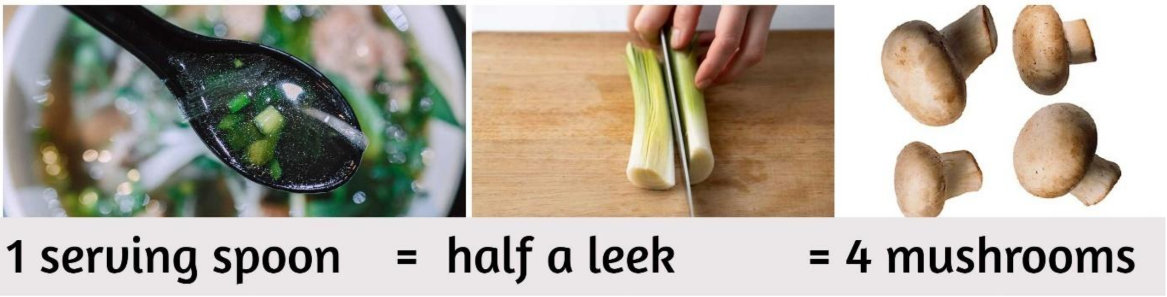

Approximately how much vegetables (except potatoes - fresh, cooked, dry) were discarded in the past week?

☐ *Less than 1 serving spoon*
☐ *1 to 2 serving spoons*
☐ *3 to 4 serving spoons*
☐ *5 to 6 serving spoons*
☐ *More than 6 serving spoons*
☐ *No part of this food item was disposed of*
☐ *I do not consume this food item*

I do not consume this food item

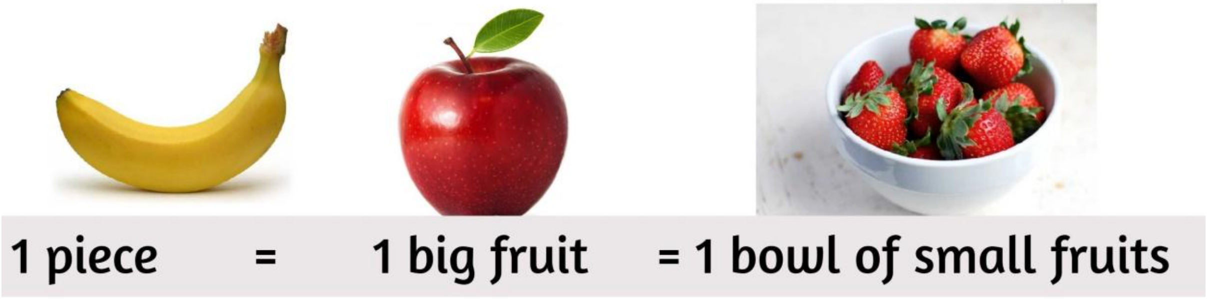

Approximately how many fruits (fresh, dry) were discarded in the past week?

☐ *One-fourth of a piece of fruit or less (a quarter)*
☐ *About half a piece of fruit*
☐ *Approximately 1 piece of fruit*
☐ *2 to 4 pieces of fruit*
☐ *More than 4 pieces of fruit*

☐ No part of this food item was disposed of
☐ I do not consume this food item

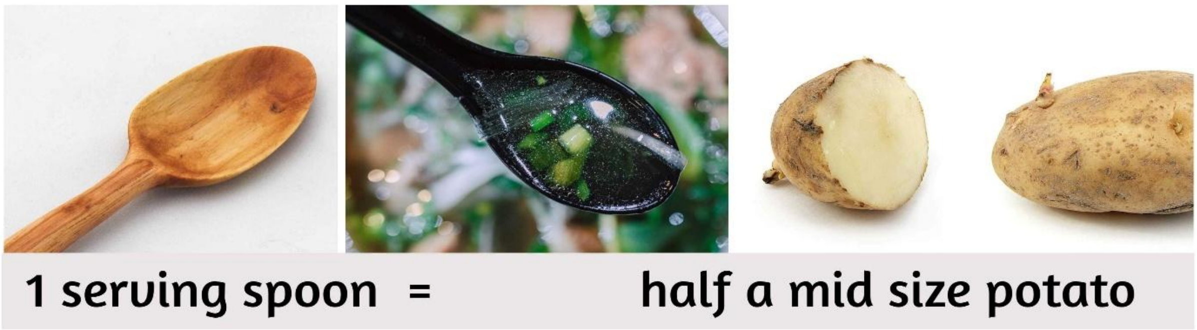

Approximately how much potatoes (fresh, cooked) were discarded in the past week?
☐ Less than 1 serving spoon
☐ 1 to 2 serving spoons
☐ 3 to 4 serving spoons
☐ 5 to 6 serving spoons
☐ More than 6 serving spoons
☐ No part of this food item was disposed of
☐ I do not consume this food itemLess than half soup ladle

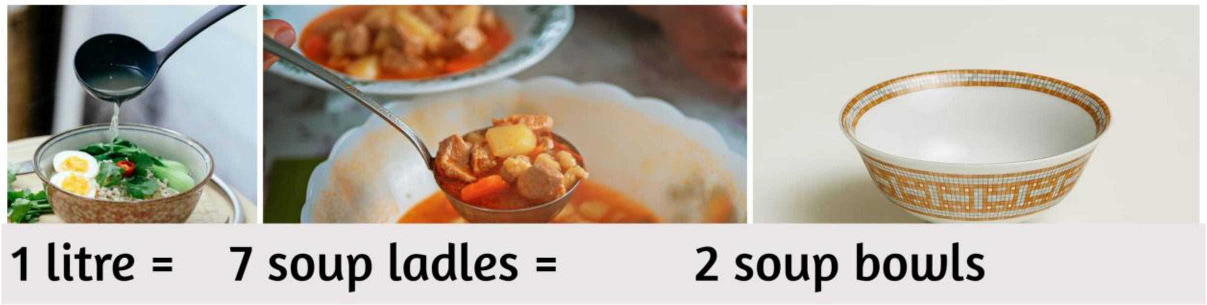

Approximately how much soup/curry/sauce were discarded in the past week?
☐ Less than half soup ladle
☐ Between half to one and a half soup ladle
☐ Multiple soup ladles (about half a litre)
☐ About 1 litre
☐ More than 1 litre
☐ No part of this food item was disposed of
☐ I do not consume this food item

Bread, Grains, and Cereals

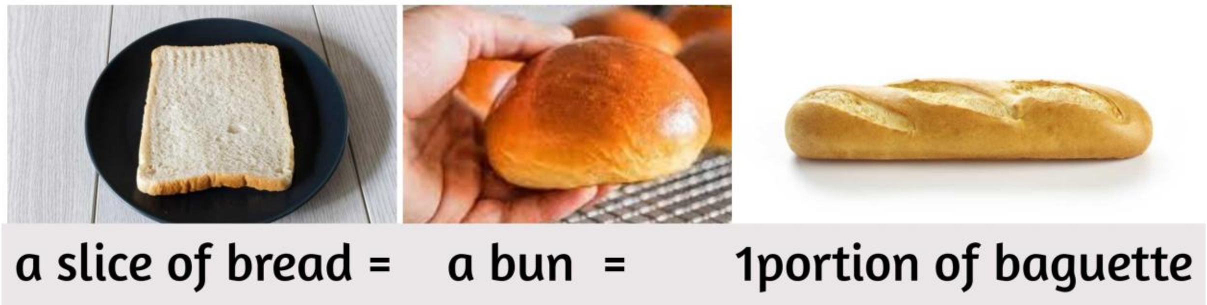

Approximately how much bread was discarded in the past week?
☐ Less than one slice of bread
☐ One or a few slices of bread
☐ About half a loaf

☐ *About one loaf*
☐ *More than one loaf*
☐ *No part of this food item was disposed of*
☐ *I do not consume this food item*

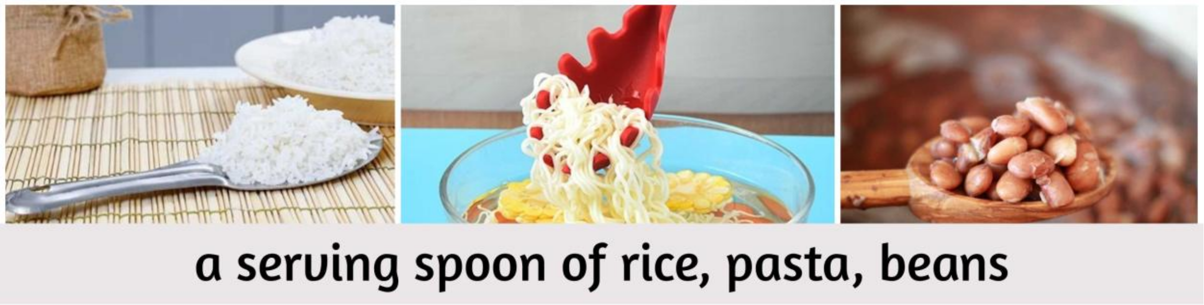

Approximately how much rice, pasta, beans, and other grains were discarded in the past week?
☐ *Less than 1 serving spoon*
☐ *1 to 2 serving spoons*
☐ *3 to 4 serving spoons*
☐ *5 to 6 serving spoons*
☐ *More than 6 serving spoons*
☐ *No part of this food item was disposed of*
☐ *I do not consume this food item*

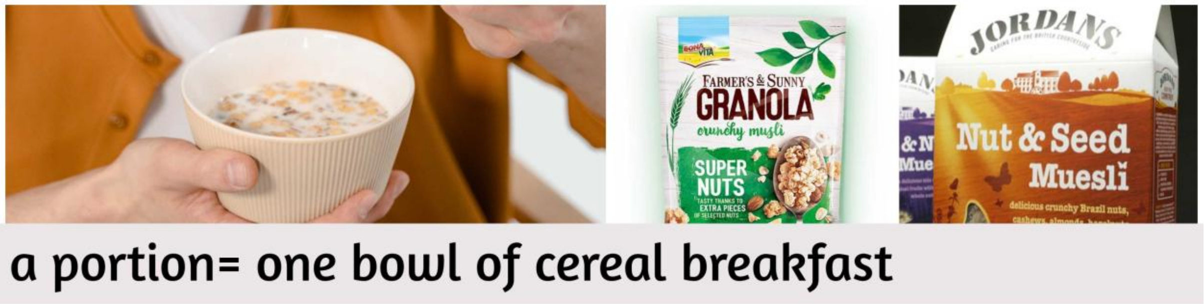

Approximately how much cereals & nuts (muesli, granola, etc.) were disposed of in the past week?
☐ *Less than half a portion*
☐ *Half to one and a half portion*
☐ *Multiple portions (about half 1 kg package)*
☐ *About 1 complete package (1 kg)*
☐ *Multiple 1 kg packages*
☐ *No part of this food item was disposed of*
☐ *I do not consume this food item*

Fish and Meat

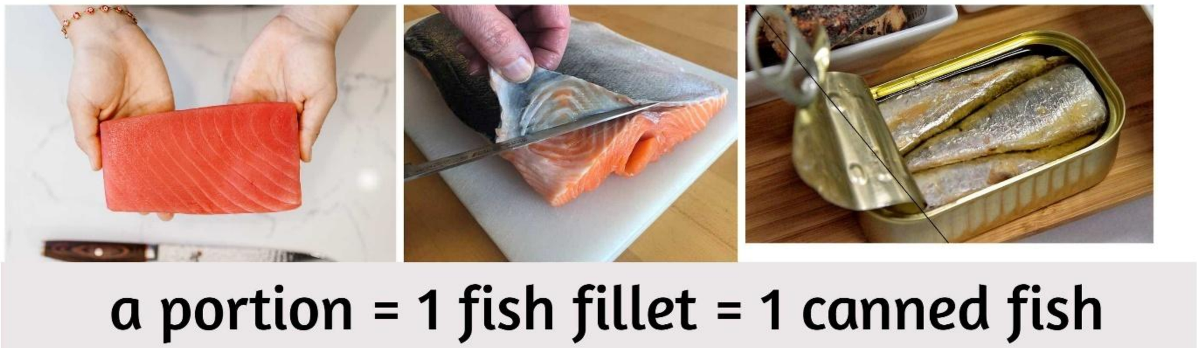

Approximately how much fish (fresh, canned, smoked, etc.) was disposed of in the past week?
☐ *About half a portion or less*

☐ *About one portion*
☐ *2 to 3 portions*
☐ *4 to 5 portions*
☐ *More than 5 portions*
☐ *No part of this food item was disposed of*
☐ *I do not consume this food item*

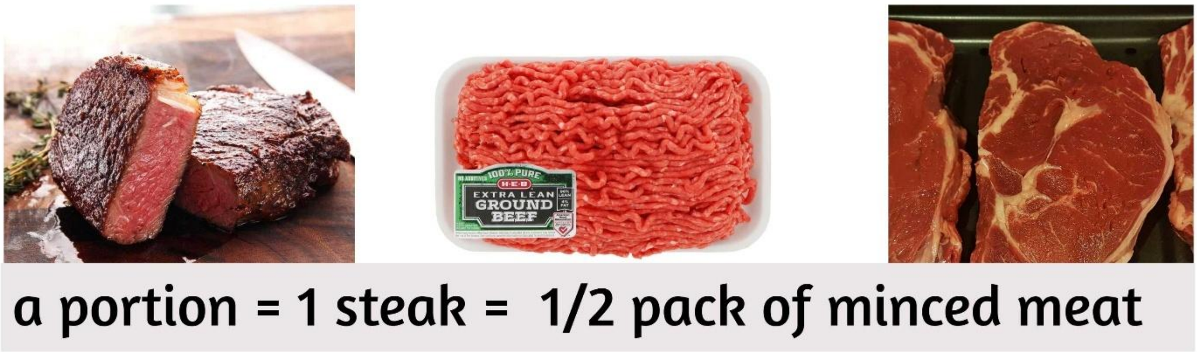

Approximately how much red meat (beef, lamb, pork, etc.) was disposed of in the past week?
☐ *About half a portion or less*
☐ *About 1 portion*
☐ *2 to 3 portions*
☐ *4 to 5 portions*
☐ *More than 5 portions*
☐ *No part of this food item was disposed of*
☐ *I do not consume this food item*

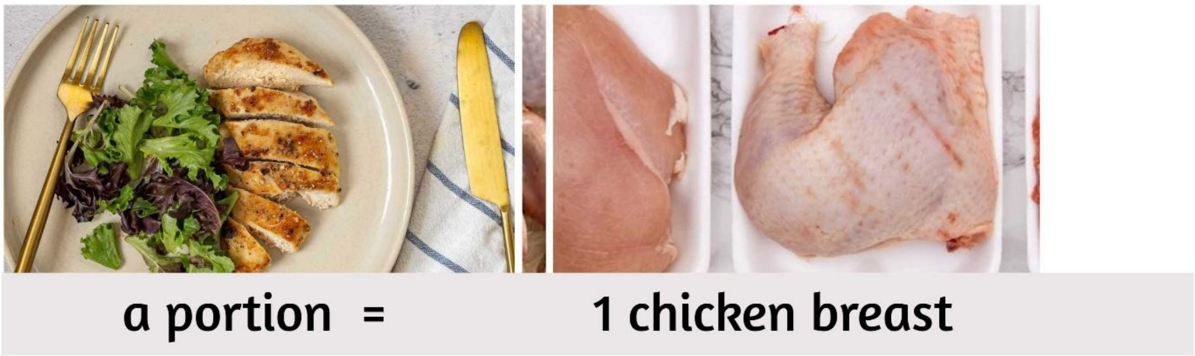

Approximately how much white meat (chicken, turkey, etc.) was disposed of in the past week?
☐ *About half a portion or less*
☐ *About 1 portion*
☐ *2 to 3 portions*
☐ *4 to 5 portions*
☐ *More than 5 portions*
☐ *No part of this food item was disposed of*
☐ *I do not consume this food item*

Egg, milk and yoghurt

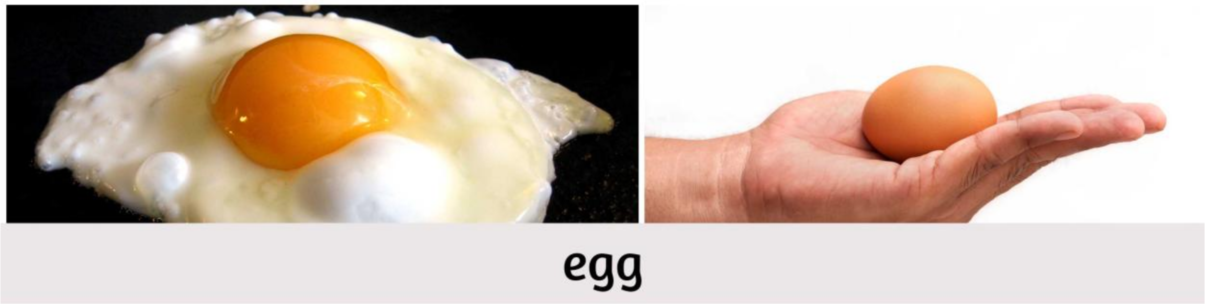

Approximately how many eggs (boiled, fried, etc.) were disposed of in the past week?
☐ Less than 1 egg
☐ 1 egg
☐ 2 to 3 eggs
☐ 4 to 5 eggs
☐ More than 5 eggs
☐ No part of this food item was disposed of
☐ I do not consume this food item

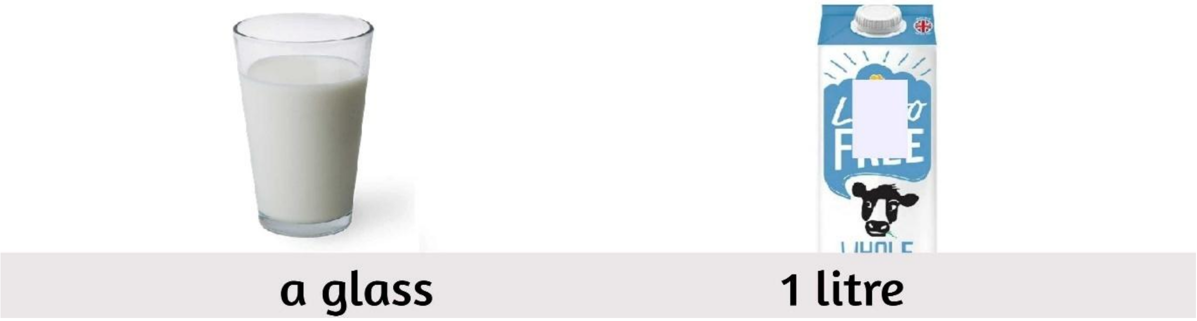

Approximately how much milk and yoghurt were disposed of in the past week?
☐ Less than half a glass
☐ Half to one and a half glass
☐ Half a litre
☐ 1 litre
☐ More than 1 litre
☐ No part of this food item was disposed of
☐ I do not consume this food item

Reasons for generating food waste and motivations for reduction

Please rank the reasons from Top to Bottom (5) 1 = once; 2 = twice; 3 = three times; 4 = four times; 5 = five or more times
In the past week, how many times did you throw away food because of the ff.

Reason	Rank (1-5)	Does not apply
I had leftovers from cooking too much	____	☐
! had cooked and served more than was eaten	____	☐
Throw away food because it had passed its best-before or use-by date	____	☐
Throw away food because it had spoiled or looked unfit to eat	____	☐
Throw some food away to clear space for new food items	____	☐

Please rank the factors from Top (1) = Highly motivated to Bottom (5) = Least motivated
In the past week, I was motivated to reduce the amount of food discarded for the following reasons

Motivation Factor	Rank (1-5)	Not motivated by this
Economic reason	____	☐
It saves time	____	☐
Family/household culture	____	☐
Environmental concerns	____	☐
Personal ethics/moral	____	☐

Kindly indicate how your discarded food is distributed to the following channels in the past week. Please ensure the numbers sum up to 100, and leave non-applicable options blank:

- General rubbish bin/mixed waste: ______
- Composting/gardening % ______
- Brown bin (organics) kitchen caddy: % ______
- Feed to pets/animals: %
- Poured down the sink% ______

Final lap, a few more questions to go! We truly appreciate your persistence.

Diet and Food Shopping

Which most accurately describes your current diet?

☐ *I eat meat, fish, and vegetables (omnivorous)*
☐ *I eat meat, but I try to limit the amount I eat (flexitarian)*
☐ *I do not eat meat, but I may or may not consume eggs or dairy (vegetarian)*
☐ *I do not eat any animal-sourced foods (vegan)*
☐ *I do not eat meat, but I eat fish and seafood (pescatarian)*
☐ *Other (please specify):* _______________

How often do you typically shop for household food items in a week?

Food Shopping and handling

☐ *Once a week*
☐ *2 times a week*
☐ *3 times a week*
☐ *4 times a week*
☐ *5 times or more in a week*

In an average week, approximately how much(€) do you spend on your household food shopping?

0 [illegible] 10[illegible] 200[illegible] ______

In an average week, how often do you engage in the following habits while shopping for food? Please skip those that do not apply to you.
0= Never 100= All the time

Buy only what is on the shopping list while shopping for food?

[illegible] 50% 100% All the time ______

In the past week, on how many of your food shopping trips did you check use-by or best-before dates before buying?

☐ *Once a week*
☐ *2 times a week*
☐ *3 times a week*

☐ *4 times a week*
☐ *5 times or more in a week*

In the past week, how many times did you engage in the following?

Activity	Ones	2 times	3times	4times	5+ times	Not applicable (0 times)
Prepare lunch at home	☐	☐	☐	☐	☐	
Prepare dinner at home	☐	☐	☐	☐	☐	
Eat takeaway meals or eat out at a restaurant, café, or similar.	☐	☐	☐	☐	☐	
Remember to use up leftovers or previously saved food	☐	☐	☐	☐	☐	
Save 'home' leftovers with intention to eat or use later	☐	☐	☐	☐	☐	

Thank you very much for participating in the survey!
