# Supplementary material for: Behavioural and Socioeconomic Drivers of Household Food Waste in Ireland: Evidence for Targeted Food Policy
Source: Foods. 2026 Aug 20;15(16):2920. doi: 10.3390/foods15162920 (PMC13512863; doi:10.3390/foods15162920)
Supplement: Supplementary file 1 [file foods-15-02920-s001.zip › Supplementary Tables and figures.pdf]

Table S1. First-stage residualisation model and residual diagnostics (N = 966).

Parameter / Statistic	B / Value	SE	t	p	95% CI
**Model coefficients**					
Intercept	265.50	58.59	4.53	< 0.001	[150.52, 380.48]
Household size	193.50	17.24	11.23	< 0.001	[159.67, 227.33]
**Residual distribution**					
n	966				
Median	0.0				
Mean	226.8				
SD	811.3				
Minimum	−1,177				
Maximum	+4,414				
Skewness	1.72				
Kurtosis (excess)	3.69				
Negative values (%)	50.2				
Shapiro–Wilk	W = 0.79			< 0.001	

Note. Dependent variable: total household food waste (g household^{-1} week^{-1}). Household size entered as a continuous variable (number of persons, 1–8). Model pseudo-R^2 = 0.088. Expected household waste = 265.50 + 193.50 × household size; each additional household member is associated with an increase of approximately 194 g in the typical household's weekly food waste. The numbers of adults and children were collapsed into a single household-size variable because their per-person contributions were statistically indistinguishable (Wald test, $F(1, 963) = 0.20$, $p = 0.657$). Residual food waste = observed − expected waste; positive values indicate waste above the size-based expectation. The right-skewed, non-normal residual distribution is the reason the second-stage analysis employs median regression, which is robust to non-normality and to the upper tail.

Table S2. Bivariate correlations between household food waste drivers and weekly waste volumes

Variable	Correlation (ρ)	p-Value
Best before date waste	0.605	<0.001
Grocery spending	0.583	<0.001
Shopping frequency	0.467	<0.001
Mixed waste disposal	0.422	<0.001
Cultural motivation reduction	0.299	<0.001
Environmental motivation reduction	0.282	<0.001

Ethical motivation reduction	0.221	<0.001
Non-home meal consumption frequency	0.206	<0.001
Spoiled food waste	0.200	<0.001
Leftover saving frequency	0.173	<0.001
Food waste poured down sink	0.024	0.448
Food waste fed to pets	0.010	0.764
Expiry date checking frequency	−0.061	0.058
Composted food waste	−0.068	0.035
Lunch preparation frequency	−0.094	0.003
Dinner preparation frequency	−0.106	<0.001
Organic waste bin	−0.163	<0.001
Leftover-induced waste	−0.234	<0.001
Storage space-induced waste	−0.262	<0.001
Excess cooking waste	−0.276	<0.001
Leftover reuse frequency	−0.290	<0.001
Shopping list usage frequency	−0.309	<0.001
Time-saving motivation reduction	−0.352	<0.001
Economic motivation reduction	−0.566	<0.001

Table S3. Weekly household food waste volumes by sociodemographic, geographic, and dietary factors in the Republic of Ireland

Variable/Level	Mean	Median	p-Value
**Age**			**<0.001**
18–24 years	305.07	203.62	
25–34 years	127.94	−163.74	
35–44 years	2.44	−129.88	
45–54 years	−94.68	−313.48	
55–64 years	−181.37	−323.48	
65 years or older	−211.47	−284.82	
**Household income**			**0.013**
< €20,000	−193.44	−456.90	
€20,000–€40,000	107.10	−138.74	
€40,000–€60,000	13.68	−161.74	
€60,000–€80,000	−68.97	−299.14	

€80,000–€100,000	141.31	−99.90	
> €100,000	−83.43	−278.06	
**Irish/Non-Irish**			**<0.001**
Irish	130.78	−89.90	
Non-Irish	−202.56	−352.74	
**Educational level**			**0.383**
Up to vocational	32.65	−275.40	
Undergraduate degree	22.33	−148.48	
Master's & PhD	−49.66	−263.80	
**Employment**			**0.038**
Full-time	−19.87	−250.38	
Part-time	83.01	−99.74	
Retired	−206.78	−364.22	
Unemployed	172.22	−163.74	
**Province**			**0.505**
Dublin	18.53	−199.74	
Leinster (excluding Dublin)	−5.65	−274.06	
Connacht	−72.55	−147.74	
Munster	−37.03	−247.64	
Ulster	193.03	−21.22	
**Settlement type**			**<0.001**
More rural	−117.27	−258.38	
More urban	75.06	−186.38	
**Dietary patterns**			**<0.001**
Flexitarian	225.77	374.90	
Omnivorous	159.31	−46.64	
Vegan	−468.61	−572.74	
Vegetarian	−513.77	−606.72	

Table S4. Hierarchical quantile regression results predicting weekly household food waste generation (Base Model 1; main effects).

**Variables**	**B**	**SE**	**Sig.**	**95% CI**
Hierarchy 1: Conditions Prompting Discard	***MAE = 435.52; R^2 = 0.239***			
Best before date waste	238.80	8.45	<0.001	[222.21, 255.39]
Food waste due to spoilage	93.08	8.69	<0.001	[76.02, 110.14]
Hierarchy 2: Motivational Factors	***MAE = 400.03; R^2 = 0.301***			
Economic motivations	−94.28	9.25	<0.001	[−112.43, −76.13]
Time-saving motivations	−124.25	9.17	<0.001	[−142.24, −106.26]

Hierarchy 3: Shopping Behaviours	***MAE = 333.40; R^2 = 0.418***			
Shopping frequency	153.50	13.19	<0.001	[127.59, 179.42]
Grocery spending	0.81	0.24	0.001	[0.33, 1.29]
Shopping list use	−41.36	9.71	<0.001	[−60.45, −22.28]
Hierarchy 4: Meal Management	***MAE = 326.31; R^2 = 0.430***			
Non-home meal frequency	65.53	16.41	<0.001	[33.35, 97.70]
Leftover saving frequency	39.84	12.23	0.001	[15.85, 63.84]
Leftover reuse frequency	−52.93	12.11	<0.001	[−76.68, −29.18]
Hierarchy 5: Disposal Methods	***MAE = 318.50; R^2 = 0.443***			
General mixed waste	6.38	0.48	<0.001	[5.43, 7.33]
Organic waste bin usage	1.79	0.48	<0.001	[0.86, 2.72]
Hierarchy 6: Sociodemographics	***MAE = 314.94; R^2 = 0.450***			
Dietary patterns (Ref: Omnivorous)				
Flexitarian	−75.60	36.24	0.037	[−146.68, −4.53]
Vegetarian	−125.96	44.89	0.005	[−214.00, −37.93]
Household income (Ref: > €100,000)				
€20,000–€40,000	189.96	54.05	<0.001	[83.80, 296.12]
€40,000–€60,000	101.68	51.58	0.049	[0.30, 203.07]
Education (Ref: Undergraduate)				
Master's/PhD	−85.47	43.56	0.050	[−170.90, −0.04]
Ethnicity (Ref: Irish)				
Non-Irish	−71.97	26.76	0.007	[−124.60, −19.33]

Table S5. Hierarchical quantile regression results predicting weekly household food waste generation (Base Model 2; interactions only).

**Variable**	**B**	**SE**	**Sig.**	**95% CI**
Conditions Prompting Discard	***MAE = 478.66; R^2 = 0.164***			
Best-before-date × Age (Ref. 45–54)				
18–24 yrs	111.85	17.87	<0.001	[76.78, 146.91]
25–34 yrs	79.72	14.07	<0.001	[52.11, 107.33]
55–64 yrs	36.00	16.93	0.034	[2.78, 69.22]
Food waste due to spoilage × Household income (Ref: > €100,000)		***MAE = 397.41; R^2 = 0.306***		
< €20,000	69.82	24.38	0.004	[21.98, 117.67]
€20,000–€40,000	113.61	15.66	<0.001	[82.88, 144.33]
€40,000–€60,000	98.71	16.22	<0.001	[66.87, 130.54]
€60,000–€80,000	53.53	16.80	0.001	[20.57, 86.50]
€80,000–€100,000	72.71	14.00	<0.001	[45.24, 100.18]
Best-before-date × Settlement type	***MAE = 360.03; R^2 = 0.371***			
Urban	101.49	10.36	<0.001	[81.16, 121.83]

Shopping Behaviours	***MAE = 321.07; R^2 = 0.439***			
Shopping frequency × Household income (Ref: > €100,000)				
< €20,000	108.51	43.18	0.012	[23.77, 193.24]
€20,000–€40,000	187.84	24.68	<0.001	[139.40, 236.28]
€40,000–€60,000	90.87	13.04	<0.001	[65.29, 116.46]
€60,000–€80,000	43.00	15.14	0.005	[13.28, 72.72]
€80,000–€100,000	156.78	21.51	<0.001	[114.56, 199.00]
Meal Management	***MAE = 312.51; R^2 = 0.454***			
Leftover reuse frequency × Age (Ref 45–54)				
18–24 yrs	−52.53	26.81	0.050	[−105.14, −0.08]
25–34 yrs	−57.77	20.12	0.004	[−97.26, −18.27]
55–64 yrs	−115.61	23.04	<0.001	[−160.82, −70.40]
Leftover reuse frequency × Household income (Ref: > €100,000)				
< €20,000	−57.21	29.02	0.049	[−114.17, −0.26]
€20,000–€40,000	−21.69	17.58	0.033	[−56.20, −12.82]

Table S6. Behavioural and psychological predictors: item wording, response options, and coding

Variable	Questionnaire question	Response options and codes	Unit
Leftover-induced waste	"In the past week, how many times did you throw away food because you had leftovers from cooking too much?"	Not applicable (0 times); 1 = once; 2 = twice; 3 = three times; 4 = four times; 5 = five or more times	Occasions per week (0–5+)
Excess cooking waste	"In the past week, how many times did you throw away food because you had cooked and served more than was eaten?"	Not applicable (0 times); 1 = once; 2 = twice; 3 = three times; 4 = four times; 5 = five or more times	Occasions per week (0–5+)
Best before date waste	"In the past week, how many times did you throw away food because it had passed its best-before or use-by date?"	Not applicable (0 times); 1 = once; 2 = twice; 3 = three times; 4 = four times; 5 = five or more times	Occasions per week (0–5+)
Spoiled food waste	"In the past week, how many times did you throw away food because it had spoiled or looked unfit to eat?"	Not applicable (0 times); 1 = once; 2 = twice; 3 = three times; 4 = four times; 5 = five or more times	Occasions per week (0–5+)
Storage space-induced waste	"In the past week, how many times did you throw away food to make space for new items?"	Not applicable (0 times); 1 = once; 2 = twice; 3 = three times; 4 = four times; 5 = five or more times	Occasions per week (0–5+)

Economic motivation reduction	"In the past week, I was motivated to reduce the amount of food discarded for economic reasons	Reverse-coded: 5 = highly motivated … 1 = least motivated	Rank score (1–5)
Time-saving motivation reduction	"In the past week, I was motivated to reduce the amount of food discarded because it saves time."	Reverse-coded: 5 = highly motivated … 1 = least motivated	Rank score (1–5)
Cultural motivation reduction	"In the past week, I was motivated to reduce the amount of food discarded because of family or household culture	Reverse-coded: 5 = highly motivated … 1 = least motivated	Rank score (1–5)
Environmental motivation reduction	"In the past week, I was motivated to reduce the amount of food discarded because of environmental concerns." .	Reverse-coded: 5 = highly motivated … 1 = least motivated	Rank score (1–5)
Ethical motivation reduction	"In the past week, I was motivated to reduce the amount of food discarded because of personal ethics or morals." .	Reverse-coded: 5 = highly motivated … 1 = least motivated	Rank score (1–5)
Shopping frequency	"In the past week, how many times did you shop for household food items?"	Not applicable (0 times); 1 = once; 2 = twice; 3 = three times; 4 = four times; 5 = five or more times	Shopping trips per week
Grocery spending	"In the past week, approximately how much (€) did your household spend on food shopping?"	Open numeric entry, recorded to the nearest euro	€ per week
Shopping list usage frequency	"In an average week, how often do you buy only what is on your shopping list when shopping for food?" (0 = never, 100 = all the time)	Slider responses banded into five ordered categories: 1 = 0–20%; 2 = 21–40%; 3 = 41–60%; 4 = 61–80%; 5 = 81–100%	Band (% of shopping occasions)
Date checking when purchasing	"In the past week, on how many of your food shopping trips did you check use-by or best-before dates before buying?"	Not applicable (0 trips); 1 = once; 2 = twice; 3 = three times; 4 = four times; 5 = five or more times	Trips per week
Lunch preparation frequency	"In the past week, how many times did you prepare lunch at home?"	Not applicable (0 times); 1 = once; 2 = twice; 3 = three times; 4 = four times; 5 = five or more times	Occasions per week
Dinner preparation frequency	"In the past week, how many times did you prepare dinner at home?"	Not applicable (0 times); 1 = once; 2 = twice; 3 = three times; 4 = four times; 5 = five or more times	Occasions per week

Non-home meal consumption frequency	"In the past week, how many times did you eat a takeaway meal or eat out at a restaurant, café or similar?"	Not applicable (0 times); 1 = once; 2 = twice; 3 = three times; 4 = four times; 5 = five or more times	Occasions per week
Leftover saving frequency	"In the past week, how many times did you save leftovers with the intention of eating or using them later?"	Not applicable (0 times); 1 = once; 2 = twice; 3 = three times; 4 = four times; 5 = five or more times	Occasions per week
Leftover reuse frequency	"In the past week, how many times did you use up leftovers or previously saved food?"	Not applicable (0 times); 1 = once; 2 = twice; 3 = three times; 4 = four times; 5 = five or more times	Occasions per week

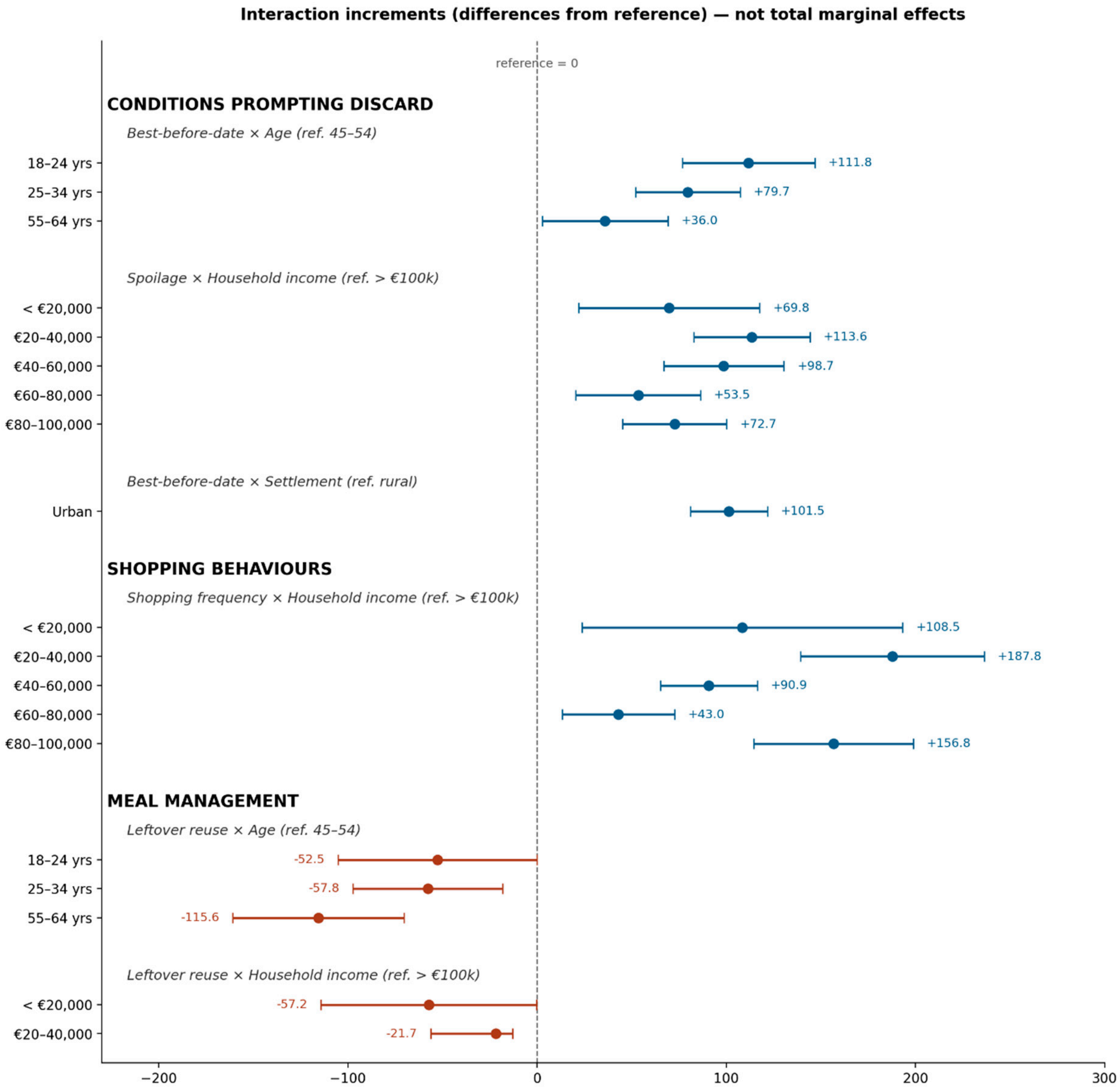

Figure S1. Interaction Coefficients with 95% Confidence Intervals Plot
